# Inside the world of non-suicidal self-injury e-communities: Language, identity and need for belonging

**DOI:** 10.1371/journal.pone.0339975

**Published:** 2025-12-31

**Authors:** Vinay Jagdish Sukhija, Elisa Mancinelli, Rachele Del Guerra, Silvia Salcuni

**Affiliations:** 1 Department of Developmental and Socialization Psychology, University of Padova, Padova, Italy; 2 Department of Surgery, Medicine, Dentistry and Morphological Sciences with Transplant Surgery, Oncology and Regenerative Medicine Relevance, University of Modena & Reggio Emilia, Modena, Italy; Universidade Federal do Rio Grande do Sul, BRAZIL

## Abstract

Non-suicidal self-injury e-Communities are increasingly gaining popularity, and people who self-harm are turning to these groups to share their experiences and feelings. They are doing so through a unique set of specific slang words related to the behaviour of self-harm that seems to be pertinent to these e-communities. In this regard, this study aims to explore slang words and differences in their usage across communities. A sample of 410 posts and respective comments were extracted from two self-harm e-communities on Reddit based on predetermined slang keywords through Python Reddit API Wrapper. A content analysis was performed, indicating that slang words prevailed across 8 different domains; the 3 most prevalent were *sense of belonging, medical care,* and *sarcasm and self-deprecation.* Inter-rater reliability of the analysis found strong agreement across the 3 individual coders. Chi-square analyses were then performed to evaluate differences in the frequency of domains and subdomains between the two self-harm e-communities. Significant differences were observed across subdomains *(X*^*2 *^*= 244.9, p = 0.001)* but not across the domains. Finally, sentiment analysis was conducted, and Mann-Whitney U-tests across the two communities found that one of the two is significantly more negative in sentiment value *(U = 23808, p = 0.019)* while, consequently, the other had a significantly larger overall compound sentiment score *(U = 17429, p = 0.003)*. Overall, lived experience findings from the textual descriptions of users indicate the pervasiveness of slang words across these communities and the further need to investigate their nuanced and varied usage. Building on the person-centred framework in NSSI research, a case is made for the development of more targeted and tailored interventions, such as e-health mobile and application-based interventions, that consider the unique contributions of NSSI e-Communities in the life and context of a person who engages in self-harm.

## 1. Introduction

Non-Suicidal Self-Injury (NSSI) is a behaviour predominantly undertaken by adolescents and young adults [1], and it can serve multiple functions such as alleviating negative states, inducing positive states, or meeting an interpersonal demand [2,3]. NSSI is commonly defined as the direct, intentional harm to one’s own body tissue without the need to die for purposes that are not accepted socially or culturally [4–6]. The age of onset of this behaviour tends to vary across different studies, with a common window being that of early adolescence, through ages of 11–15 [7]. While there is some evidence that indicates the onset of NSSI before puberty [8], and another study indicating that an earlier onset is linked to relatively higher frequency and more severe methods [9], this behaviour typically tends to peak through ages of 15–17 [10]. Understanding, tackling, and reducing the behaviour of NSSI is a growing public health concern, especially since evidence points toward the growing prevalence rates succeeding the COVID-19 pandemic [11].

The four-functional model used to explain the functions of NSSI hypothesize this behaviour across two dimensions; serving automatic regulation or serving social regulation. This regulation could be achieved through both positive and negative reinforcements, thus arriving at four different functions that NSSI is typically found to serve. Therefore, individuals could use it to generate emotion and stimulation (automatic positive reinforcement), reduce tension (automatic negative reinforcement), signal distress and seek attention (social positive reinforcement), and avoid interpersonal demands (social negative reinforcement) [2]. Another theoretical model, known as the “Experiential-Avoidance Model” posits that NSSI is predominantly sustained by the negative reinforcement of escaping undesirable negative emotions [12]. Moreover, earlier research has also discussed NSSI as a form of distress signalling; researchers have hypothesized that the act of NSSI, due to its costly nature (both physical and emotional costs), is more likely to allow the person to be taken seriously since it serves as a more intense signal in social contexts than just language. One of the goals of this signalling is hypothesized to be that of receiving caregiving behaviour from an individual’s environment [13]. In light of these theoretical backgrounds, it could be assumed that people who self-harm look toward digital media and the online world both to escape undesirable emotions and elicit caregiver behaviour through sharing of experiences.

Users typically tend to turn to memberships in e-Communities where people get together and share their narratives and interact with each other to discuss experiences centred around a certain theme or a shared commonality [14]. In this regard, NSSI e-Communities are a relatively new phenomenon, where users get together and share their experiences, or seek knowledge and advice online from each other related to the behaviour of NSSI [15–18]. Data show that there is a consistently growing membership to these NSSI e-Communities, which are spread across multiple platforms such as Instagram, Twitter, TikTok, Reddit, and Tumblr [16,19–22]. This is particularly relevant given that evidence points toward the fact that exposure to increasingly severe material related to self-harm on the internet led to other people responding with an increased number of comments [23] and that the sharing of similar narratives could lead to a type of ‘narrative reinforcement’ where people feel reinforced in the continuation of this behaviour [24]. Moreover, information on NSSI online contains advice and information that is seldom provided by credible sources, thus increasing the likelihood of misinformation and claims about topics that have not been corroborated [25,26]. On the other hand, there is enough evidence that points towards the benefits of these NSSI e-Communities [27]. Prior research on these e-Communities indicates how the absence of stigma, presence of pseudo-anonymity, instant access to support, availability of information, and the feeling of belonging to a community were found to be very beneficial to users of these groups [15,17,28]. Prior research has found that people who self-harm tend to join these communities for multiple reasons, such as seeking support, understanding NSSI, and getting or offering NSSI help [29]; for instance, one research article also found that users who engage in these e-Communities tend to decrease their NSSI behaviour over time [30]. Moreover, it was also observed that members claimed to maintain their membership in these communities since they valued the relationships and camaraderie they built in these groups [29]. Another study then described user behaviour on NSSI e-Communities as a form of anonymous-public display of NSSI, where identity secrecy can be maintained, while simultaneously publicly sharing experiences, most likely for the above-mentioned benefits [31].

For researchers, on the other hand, one benefit of NSSI e-Communities is rich access to people’s lived experiences that have long been a difficult variable to incorporate into self-harm research [32]. One article even suggests that access to these detailed and unfiltered descriptions of people’s self-harm behaviours could provide researchers with unique information that could allow them to reconceptualize NSSI interventions with a higher efficacy [27]. One aspect of these lived experiences is the usage of slang by members of these communities. Slang has been a topic of interest in linguistic research for a long time. Slang can be defined as the improper, unsystematic, and informal language that diverges from standard lexicon, and typically stems from a transgressive or rebellious attitude [33]. Sociologically, the purpose of slang is found to reinforce group identity and strengthen cohesiveness, which can further quicken group acceptance, facilitate friendliness, and preserve in-group solidarity [34]. In the context of self-harm, one study found the usage of slang and argot on Tumblr self-harm communities to be widely diffused. Indeed, they found the use of in-group terminology that would not have much meaning for people outside the communities of NSSI practice. The authors suspect this terminology to function as codewords intended to conceal NSSI activity and maintain secrecy, a common function of slang. For instance, one article that focused on NSSI posts on Instagram studied 201 Instagram posts and found the use of ambiguous NSSI hashtags where the unequivocal meaning of NSSI was not apparent. For example, the authors found self-harm photos to be accompanied by hashtags such as “Blithe”, “MySecretFamily” and “Secretsociety123”. The authors speculate, based on prior literature, that such hashtags could, in particular, enable teens who engage in this behaviour to access and feel connected to NSSI-related communities [19]. Not surprisingly, the ambiguity in language did not restrict itself to posts on Instagram, another study focused on posts of NSSI on the platform TikTok and they found similar trends. Specifically, they observed that content creators altered specific NSSI words to avoid violating the community guidelines [21]. The creators used methods such as abbreviations (e.g., SH instead of self-harm), usage of symbols instead of letters (e.g., self-h@rm instead of self-harm), and the alteration of complete words or phrases (e.g., barcode instead of scars). Thus, leading them to analyse the sociotechnical context surrounding the data using qualitative content analysis. This allowed the researchers to understand community-specific social information practices, such as experiences and language particular to that community. Accordingly, the usage of ambiguous language, argot, and slang might represent a practice of shared meaning-making, and becoming a member of a NSSI e-Community would require one to familiarize with the community-specific language [22].

Reddit, another social media forum, contains multiple NSSI e-Communities that are very popular. This platform allows people to make posts and comments in a pseudonymous method, and hosts these ‘threads’ (combination of posts and comments) on topic-driven forums known as ‘subreddits’. Two such subreddits, r/selfharm and r/MadeofStyrofoam are the most popular NSSI e-Communities with memberships of 156,000+ and 78,500 + respectively. These two communities differ significantly based on their publicly available “Community Rules”. Specifically, r/selfharm was a community with a much larger number of memberships and subsequently contained a larger number of group moderators. These moderators voluntarily engaged in cleaning the community and deleting/censoring posts that did not adhere to the guidelines of this community. The guidelines were provided in technical and formal language and included rules such as the prohibition of uploading photos of one’s self-harm, seeking medical advice, glorification of self-harm, providing elaborate instructions on self-harm, and encouraging self-harm or suicide. In contrast, r/MadeofStyrofoam was created with the belief that people who self-harm ought to have the ability to express themselves more freely. In this regard, there was far less moderation and censorship of the group. The community still had guidelines that they adhered to, but were far more lenient in enforcing them. Moreover, the guidelines were written in an informal language and even contained the use of swear words. Guidelines included the prohibition of sharing photos of self-harm, photos of tools used to self-harm, glorification of self-harm, encouraging anyone to self-harm, and claiming themselves as more valid because they engage in more severe self-harm.

As highlighted above, NSSI e-Communities provide an interesting field site to observe peer support dynamics and the influence of peer interactions on the behaviour of self-harm. Moreover, these sites allow researchers to understand trends or novel developments regarding this behaviour due to the increased likelihood of people sharing their experiences openly in ways that they would perhaps not be comfortable doing offline. While some studies have found social and peer support advantages of NSSI e-Communities, other studies have found the reinforcing and triggering capabilities of the same communities [35]. This study hence arose out of multiple reasons, prior literature indicating the range of functionalities NSSI e-Communities serve members on NSSI, the usage of ambiguous slang in these communities, the aptness of the qualitative content analysis method to study the sociotechnical contextual information within the communication of NSSI, and the dearth of studies on Reddit NSSI e-Communities with a special focus on the usage of slang. To the best of our knowledge, no study has attempted to explore microscopic differences across different NSSI e-Communities or the usage of slang on them. Indeed, even the subreddit name “Styrofoam” is used as a slang word that represents the “white dermis layer of skin resembling styrofoam”. Knowledge of these aspects would allow us to determine the unique ways in which these groups have the potential to be advantageous and/or disadvantageous. Accordingly, this study intended to analyse the usage of slang in the two above-mentioned e-Communities; three specific research aims were proposed: (1) to explore and determine the predominant slang words used across the two e-Communities; (2) to capture the range and quality of themes/contents that contain usage of slang words; (3) to determine differences between the two e-Communities in the usage of slang words and the context in which they appear. In this regard, given the above-reported difference in the e-Communities’ “Community Rules”, it is expected that the adoption and usage of slang words between the two will differ. The direction of such differences and kinds of differences will be exploratorily investigated.

## 2. Method

### 2.1. Procedure and sample

Reddit communities r/selfharm (C1) and r/MadeofStyrofoam (C2) were considered because they host the greatest number of memberships for subreddits centred around the topic of NSSI. To identify an appropriate sample size for our aims and ensure that they contained the most prevalent slang words, we first began by creating a list of slang words that were used by users in the two target communities. More specifically, in line with the procedure adopted by Guccini & McKinley [22] when investigating NSSI communities on Tumblr, some of the slang words were not decodable until we came across a post on one of the Reddit communities that attempted to provide definitions for the most popular NSSI-slang words. Through the above-mentioned post, a list of 16 slang words was prepared, and then a Python script was written to determine the 5 most frequently used words across the 16. Using the Python Reddit API wrapper (PRAW), frequency counts for the 16 words across the two communities (i.e., C1 and C2) were determined for the recent 1000 posts on each community, checked on February 3rd, 2023. Following this, another script was written, and using PRAW, all threads that contained one or more of the 5 most frequent slang words over a period of 3 months (November 2022-January 2023) were downloaded and written onto CSV files. Two separate files were created, one for each community.

Note that this study was not preregistered; given its exploratory and inductive nature, preregistration was not pursued, consistent with research recommendations that state preregistration could inhibit the possibility of capturing the diversity and richness of ‘lived experiences’ [36].

Overall, a total of 239 posts were downloaded from C1, and 241 posts were downloaded from C2. During the data cleaning process, 28 posts were removed from C1 and 42 from C2. Posts were excluded for one of two reasons: either the downloaded post body was void and only contained a title, or the slang word was used in its literal sense (e.g., butterfly when referring to an actual butterfly). Eventually, 410 sample units were considered for overall content analysis and sentiment analysis; 211 units were from C1 and 199 from C2, where one unit consisted of the Post Title, the Post Body, and the Post comments and replies. The total number of comments on C1 were 1684, and for C2 were 2922.

The sample units that were downloaded were analysed using conventional content analysis. The data was manually coded by three independent coders in multiple stages (VJS, EM, RDG). First, all 3 coders individually analysed all 410 units to immerse themselves in the data and familiarize themselves with them. Following this, during the second stage, all three coders classified the quality of the sample units, each coder arrived at initial interpretations for the units independently and together discussed recurring patterns and domains to arrive at an initial codebook. This codebook was then applied to the dataset together, and through analysis of every unit, codes were refined and improved to achieve a strong inter-rater reliability of at least 90% agreement on the complete sample, and disagreements were resolved through discussion and deliberation. Conventional content analysis was preferred as existing theory on this phenomenon was limited. Moreover, as prior literature recommends [37], preconceived categories were avoided in order to allow the codes and categories to flow from the text by facilitating complete immersion into the dataset.

### 2.2. Data analysis

Following the described procedure, frequency counts for all assigned domains and subdomains were determined, and Chi-square tests were performed through RStudio to assess differences in the frequencies for the domains and the subdomains across C1 and C2. Since the coding framework was inductively developed, Chi-square tests were conducted on both the domains and subdomains to capture fine-grained differences that may have otherwise been obscured if focusing solely on the broader domain level. Prior research has noted that content analysis often emphasizes subcategories as the level at which information is summarized, and therefore, we examined information from this detailed perspective as well [38].

Lastly, a sentiment analysis on the post title and body was conducted as a unit. In order to conduct the sentiment analysis, the Post Title and the Post Body of each post were combined and then the sentiment of each unit was assessed. This was run independently for each community (i.e., C1 and C2) using the Natural Language Toolkit (NLTK) package provided by Python [39]. The analysis is run by comparing the sample to a list of sentiment-laden words (lexicons) and their respective sentiment values. The analysis produces 3 different sentiment values, ranging from 0–1 of how positive, negative, and neutral the text is; these are then interpreted as a percentage of sentiment features. Hence the scores on these three values would sum up to 1. A 4^th^ overall compound score is provided which is a normalized and weighted score ranging from −1 to + 1 representing whether the paragraph leans more negative or positive. The compound score is calculated by aggregating the valence scores of each word in the corpus (positive, negative, neutral), adjusting for contextual elements and heuristics (punctuation, negation, capitalization, etc). and normalizing from a range of −1 to +1 [39].

Following this, potential differences across the sentiment values and the overall compound sentiment of the two communities were investigated through a Mann-Whitney U test on IBM SPSS Statistics for Windows, Version 28.0.1.1.

### 2.3. Ethical concerns

All data collected was publicly available and in the public domain. Moreover, all terms and conditions of the social media platform were complied with during data collection. Although Reddit is a pseudonymous platform (individuals can be identified via their username, but their original identity is not disclosed); nonetheless, care was also taken not to store any of the usernames from the posts. Moreover, all example quotes used in the following sections are paraphrased in order to prevent back-tracking to the original source. Best practices for internet research were maintained throughout, and no attempts were made to interact with users or re-identify them. Thus, keeping these considerations in mind, all data used was under the principle of free use while respecting the regulations of the GDPR. Furthermore, it should be noted that, in line with the above, the study was deemed exempt from formal ethical review from our institutional ethics board, as projects based solely on publicly available, anonymized data do not fall under the scope of mandatory review.

Due to ethical and privacy concerns, raw Reddit data cannot be shared as it may enable re-identification of users. However, the analytic material (Python code for scraping and sentiment analysis) are available upon reasonable request from the corresponding author.

## 3. Results

The 410 sample units contained an average word count of 311.05 words combined for Post title, Post Body, Post Comments and replies. Moreover, average word counts were 309.76 words and 312.34 words for C1 and C2 respectively. Additionally, across C2, 113 images/memes were found. 52 of these images were on unique threads, i.e., one image per thread. The remaining 61 images were posted on 15 different threads where each post contained multiple images ranging from 2 to 7 images per post. Because these visual materials were unique to C2, they were not included in the main cross-community comparisons. However, for transparency, we conducted a descriptive coding of these images. Four thematic domains were identified: *Representation of Self, Self-Harm Tendencies, Representation of Addiction,* and *Symbols Representing Hope.* The full descriptive coding is provided in a dedicated section in the Supplementary Material and related Table S1 in S1 File.

### 3.1. Prevalent slang words

The first research aim of this study was achieved by determining the most prevalent slang words across the 1000 most recent posts. The initial list of 16 words extracted by the recent 1000 posts allowed us to have a foundation from which frequency counts could be checked via the PRAW. Through this analysis, the 5 most prevalent slang words were: (1) styro, used 78 times (2) yeet, used 81 times (3) beans, used 80 times (4) butterfly, used 32 times and (5) juice, used 15 times across both communities.

All the 410 sample units extracted from C1 and C2 afterward, contained one or more of the above-mentioned slang words; Table 1 contains the frequency counts and meanings for these words.

**Table 1:** List of prevalent slang words, meanings, and frequency counts.

Slang words	Meanings	Frequency counts (N)
Styro	White dermis layer of the skin resembling Styrofoam	195
Yeet	Self-injury/to self-injure	151
Beans	Hypodermis layer of the skin that resembles beans	102
Butterfly	Steri-strip that resembles a butterfly	30
Juice	Blood due to self-injury	4

Note: Words appear more than once in the threads and hence the count sums up to 482.

### 3.2. Content analysis

Through the content analysis of the 410 units (i.e., post title, post body, post comments and replies), a total of 8 domains and 31 subdomains were determined. The following tables (Table 2–3) each contain individual domains and subdomains with an example quote. Table 2 contains a list of the top 4 domains in terms of frequency counts followed by example quotes to describe each category. Similarly, Table 3 contains a list of the latter 4 categories in terms of frequency counts with respective examples.

**Table 2:** Top 4 domains in terms of frequency counts.

Domains (N)	Subdomains (N)	Example Quotes
Sense of Belonging/Community (103)	Seeking support and someone to talk to (42)	*Just hit STYRO and could use someone to talk to or a distraction.*
	Venting/Ranting (42)	*Does anyone else watch videos while YEETING? Just a little vent……*
	Sharing triggering experiences (9)	*I just heard my coworkers say that I’m inefficient, I can’t wait to go home and YEET.*
	Cry for help (7)	*I’m experiencing intense urges and need to YEET. Please help me, I don’t know what to do.*
	Experiencing traumatic situations (3)	*I was bullied while having lunch at school. Went to the bathroom and hit STYRO, it bled quite a bit.*
Medical Care and Harm Management (102)	Wound closure advice (55)	*I just YEETED and I’m wondering whether it’s safe to clean the wound with isopropyl alcohol instead of soap and water?*
	Infection advice (26)	*I used toilet paper to clean my STYROS. Is there a risk of infection?*
	Care for wound with inadequate supplies/without going to hospital or ER (11)	*I only have a handful of bandages. How can I patch up STYROS?*
	Advice on engaging in potentially harmful activities (10)	*I YEETED on my right thigh, probably STYRO. Any tips on how to make running less painful?*
Sarcasm and Self-Deprecation (67)	Irony (35)	*lol no money to purchase band aids but I YEETED anyway. I’m so stupid.*
	Seeking attention (25)	*Using my YEET JUICE to draw clown makeup on my face.*
	Feelings of worthlessness and helplessness (7)	*One email made me want to take a toaster bath so badly that I ended up YEETING.*
Exploration and Curiosity about Self-Harm (54)	Reassurance that the experience is normal (27)	*I cut to STYRO, but I don’t feel anything. There is some numbness, is this normal?*
	Understanding wound severity (23)	*Does it count as BEANS if the STYRO is deep, and I can see some fat?*
	Decoding Terminology (4)	*What do words like BEANS and STYRO mean?*

**Table 3:** Latter 4 domains in terms of frequency counts.

Domains (N)	Subdomains (N)	Example Quotes
Addiction and Relapse Cycle (26)	Urges to relapse (6)	*Can you share how you cope with the urge to YEET?*
	Seeking advice to abstain (6)	*I tend to give in when I get the urge to YEET. Does anyone have tips for distractions?*
	Feelings after relapse (6)	*I relapsed today and YEETED after staying clean for a while. The problem is, I not only enjoyed it but also felt good afterward.*
	Triggers (3)	*I just figured out what triggers me to YEET, especially since I’m feeling like it after a long time.*
	Abstaining from SH by doing other harmful activities (3)	*I wish I had a better coping mechanism than overeating to stop myself from YEETING.*
	Tolerance (2)	*Every time I reach STYRO, it only stings and doesn’t hurt as much as I expected.*
Self-Worth and Validation through Self-Harm (25)	Achievement or Milestone Perception (9)	*I feel like a failure if I don’t YEET at least every other night.*
	Severity and Frequency of Self-Harm (7)	*I’ve always felt like I’ll only be valid if I cut to BEANS, so I end up cutting deeper than usual.*
	Coping Mechanism/Emotional Regulation (6)	*I feel very good after a social interaction, but then I begin overthinking it, and it turns into a YEET session since that’s the only coping mechanism I have.*
	Sense of Relief Afterward (3)	*I only hit STYRO this time, but it felt oddly relaxing.*
Concealing and Managing Scars (25)	Strategies to conceal scars (8)	*I have new YEETS on my upper arm. What are some ways I could hide them?*
	Explaining scars to others (6)	*All my cuts are STYROS. Is there a sensible explanation for these scars? I can’t conceal them anymore.*
	Scar fading concerns (6)	*Do deep YEET scars ever fade away?*
	Commonality of scarring experience (5)	*If I place a BUTTERFLY on a STYRO, will it leave a scar?*
Negative Impact and Consequences (8)	Consequences (6)	*I YEETED a while ago and felt like shit, vomiting, nausea and fatigue. Was this a consequence of my own silly actions?*
	Dislike for slang words (2)	*Does anyone else have a dislike for terms such as STYRO, BEANS, bedrock or cat scratches? It feels like we are giving cute nicknames to very serious issues.*

Although multiple sample units contained the essence of multiple domains and subdomains, it was collectively decided that each unit would be represented by the domain and subdomains that would permeate the strongest from it. Thus, facilitating the possibility of statistical analysis and cross-community comparisons.

The most frequently occurring domain in which the slang words occurred was the one we categorized as *Sense of Belonging/Community* (n = 103). This domain contained 5 different subdomains, and the crux of these subdomains was that they centred around the sense of community. Members would note how this was the only place where they felt heard and understood, thus offering them a mode of conversation that they do not have in the offline world. For instance, several users expressed that writing in the subreddit “made them feel less alone” or described it as “the only space where I can talk openly without being judged.” Others remarked that hearing from people with similar experiences “helps me feel like I belong here,” indicating that feeling of belonging was constructed through shared understanding of similar experiences.

Following this, the next domain was the one that focused on *Medical Care and Harm Management* (n = 102). This domain contained 4 subdomains that centred around questions related to medical advice, advice on engaging in daily activities after engaging in self-harm, wound treatment, and fear of infections. It is possible that members believed that other members belonging to these communities had the authority or experiential knowledge to answer questions pertaining to medical advice. Next, the domain that was found commonly across these slang words was *Sarcasm and Self-Deprecation* (n = 67). This domain had three sub-domains where the underlying theme was always the sarcasm and self-deprecation that would cut across the post. Typically, these posts contained serious information that was often disguised by a veil of sarcasm while being inherently self-deprecating. Finally, the 4^th^ most predominant domain that was found was *Exploration and Curiosity about Self-Harm* (n = 54). This contained three subdomains and essentially members would use these posts to explore their curiosity about self-harm and broaden their knowledge about it.

The latter four domains were not as predominant as the former, but nonetheless, they contain valuable information that merit further discussion. *Addiction and Relapse Cycle* (n = 26) contained 6 subdomains where members would use the slang words to describe their self-harm behaviour as a type of addiction. Units in this domain contained multiple aspects of the addiction phenomenon such as relapse, abstinence, tolerance, etc. Following this, the domain of *Self-worth and Validation through Self-Harm* (n = 25) contained 4 subdomains. These units focused on how members of these communities considered their self-harm behaviour as an aspect of their self-worth. Moreover, their behaviour of self-harm was a means for them to receive validation and regulate their emotions. Next, the domain that stood out across slang words was that where members sought to discuss methods to *Conceal and Manage Scars* (n = 25). This contains 4 subdomains where the primary aim of the posts was to discuss methods to hide scars, excuses to tell people when someone saw the scars in the real world, concerns about whether scars would fade and to understand whether experiences with scars were common and relatable. Lastly, the domain that was least common was categorized as *Negative Impact and Consequences* (n = 8) which contained two subdomains that focused on the ramifications of their actions and their aversion for the slang words.

### 3.3. Differences in communities

The following section attempts to delineate differences across the two communities (i.e., C1 and C2) across different aspects such as frequency counts of the domains, subdomains, slang words, and the sentiment of the semantic analysis.

#### 3.3.1. Frequency counts of domains, subdomains and slang words.

**Fig 1:**
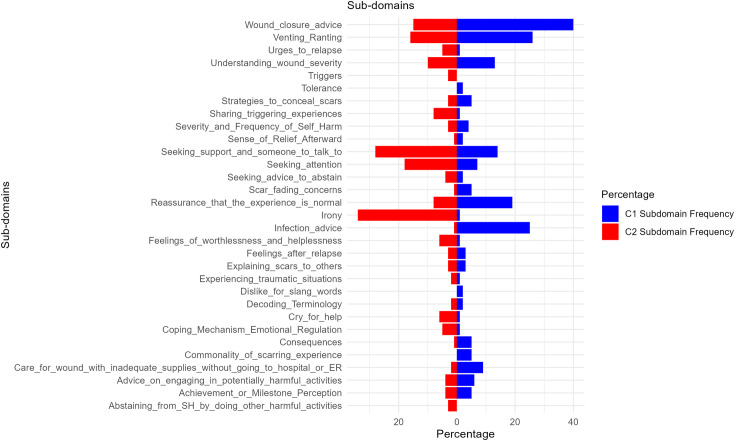
Frequency Counts of Subdomains across C1 and C2.

Owing to the mentioned differences in community rules and guidelines, it seemed imperative to assess the differences across the two communities in terms of the frequency counts for the domains, subdomains, and the usage of slang words. Results showed that C1 and C2 did not significantly differ across the frequency counts of the 5 reference slang words, or the above-mentioned 8 domains *(X*^*2 *^*= 56, p = 0.22)* (for figure of frequency counts across domains, see supplementary material in S1 File). However, there was a significant difference across the two communities in terms of the frequency counts for the 31 subdomains *(X*^*2 *^*= 244.9, p = 0.001)*. Differences in the frequencies of subdomains are delineated in Fig 1.

#### 3.3.2. Sentiment analysis.

The Natural Language Tool Kit package was used in python to arrive at sentiment scores for sample units in both communities. Following this, Mann-Whitney U test was performed to assess differences in sentiment between C1 and C2. As shown in Table 4, the two communities did not significantly differ across positive and neutral sentiment values (p values > .05). However, there was a significant difference between C1 and C2 across the negative sentiment value, where C1 had a larger negative sentiment than C2 *(U = 23808, p = 0.019)*. Consequently, C1 and C2 had a significant difference in the overall compound sentiment of the post, where C1 had a significantly lower overall compound sentiment score than C2 *(U = 17429, p = 0.003*). Visual distributions of sentiment scores are provided in Supplementary Figures S2-S5 in S1 File.

**Table 4:** Mann-Whitney (U) test sentiment score comparisons between C1 and C2.

Type of sentiment score	Community	N	Mean Rank	Mann-Whitney U	Z	p
Positive sentiment score	C1	211	203.90	20,656.500	−.283	.777
	C2	199	207.20			
Neutral sentiment score	C1	211	203.76	20,627.500	−.306	.760
	C2	199	207.34			
Negative sentiment score	C1	211	218.83	23,808.000	2.350	.019*
	C2	199	191.36			
Compound sentiment score	C1	211	188.60	17,429.000	−2.974	.003**
	C2	199	223.42			

Note. p < 0.05* and p < 0.01**

## 4. Discussion

Social media use is increasingly gaining popularity and widespread use, particularly among the younger generations [40]. The attractiveness of online NSSI e-Communities can be attributed to the fact that they offer instant connection, a pseudo-anonymous sense of safety, and advice about NSSI which a member might not have received in their real-world [41,42]. In this regard, subscriptions and memberships to NSSI e-Communities are increasingly on the rise and warrant a deeper, intricate, and nuanced exploration. In this regard, this study aimed to explore the communication of NSSI on two very popular Reddit e-Communities and explore their group-based differences.

Prior research attempting to explore ambiguous self-harm hashtags on Instagram found that these hashtags remained consistent within NSSI communities across websites, thus leading the authors to conclude that there is high motivation and commitment to creating a shared yet elusive corpus of knowledge [19]. Moreover, the authors speculate that the ambiguity of these hashtags allows followers to quickly connect with an NSSI community using phrases that have unique definitions for this group; as such, it is likely that the ambiguity is motivated by a desire to prevent recognition from outside the community, the outgroup [19]. In our study, it is evident that the usage of slang has seeped into nearly all topics of discussion and is possible that the increase in usage of this shared, yet elusive corpus of knowledge meets the same purpose of avoiding recognition from outside the community.

In this regard, the topics of discussion that were discovered in the sample units here evaluated seem to resonate with previous research that examined the needs of youth’s online posts pertaining to NSSI on a social media platform, *TalkLife* [43]. The authors found several needs that youths would exhibit while posting anything related to NSSI, and many of these themes resonated with the topics documented in this current study. For instance, similar themes include *curiosity, asking for* or *offering social support, seeking validation, asking for medical support, documenting urges,* and *hopelessness or difficult behaviours.* The authors find intricate time-based differences in needs shared by these users, and state that, in line with earlier research [42], youths’ online NSSI activity could be motivated by the fact that they feel more understood by their online peers. In this regard, it is plausible that the integration of slang words while exhibiting the above-mentioned needs could be a method to increase the feeling of being understood by peers across these communities. Literature indicates that participation in e-Communities makes members believe that they are not as different from others as they might feel, and this could then alleviate some stress and pressure from the individual [44]. This relatability with people from the online world could also be achieved by ‘narrative reinforcement’ which is the co-construction of stories to explain and justify one’s self-injury through communication with others through the usage of similar scripts [24]. It is likely that the usage of slang augments this relatability and connection among members, thus possibly reducing stress while potentially leading to the justification and rationalization of this behaviour.

The primary functions of e-Communities are twofold: to exist as a storehouse of information that members can access and to foster a spirit of community thus allowing members to develop a sense of identity [45]. In this current study, in line with expectation, the two communities C1 and C2 differed across which function tended to be more salient which is evident in the statistical differences across the subdomains. For instance, the domain *Medical Care and Harm Management* occurred 80 times on C1 and just 22 times on C2 indicating that members are more likely to seek and access medical information on the former community. During the analysis, a Google document was also found on C1 that contained elaborate medical advice on post-self-harm wound care. C1 existing as a storehouse of information has both advantages and disadvantages. For instance, individuals that engage in NSSI are viewed negatively by healthcare professionals [46], and these professionals even tend to distance themselves from patients that present frequently with this behaviour [47]. Moreover, a study found that individuals who hear other’s bad experiences with healthcare professionals tend to create a strong barrier and avoid seeking help [48]. It is likely that e-Communities such as C1 can serve as an easy and readily available location for people to access medical information in a pseudo-anonymous manner that helps them avoid the potential negative consequences of seeking health care. Indeed, the subdomain *Care for wounds with inadequate supplies/without going to hospital or ER* contained units that seemed to sidestep all of these concerns and seek medical advice in an accessible way. However, on the other hand, social media is well known to be a place that contains medical misinformation [49]. Therefore, it is possible that advice existing on C1 could be misinformed, this complemented with the fact that a person who engages in self-harm is less likely to seek medical help [50] could have negative consequences for the members.

C2, on the other hand, seemed to focus more on fostering a spirit of community and allowing members to develop a sense of belonging. For instance, the domain *Sense of Belonging/Community* occurred 60 times on C2 and 43 times on C1. We know from prior literature that *“sense of belonging”* is a crucial feature for participation in virtual communities [51]. Moreover, in the context of NSSI, prior literature suggests that the engagement and support people receive in these communities could transform into feelings of belonging and community. In this regard, the fact that the posts on C2 had a higher frequency of units categorized as *Sense of Belonging/Community* perhaps indicate that people viewed C2 as a place for building community spirit. Moreover, the effort to build a community spirit is visible not just by members but also by group moderators due to the creation and implementation of an automatic comment bot that would leave a comment titled *‘Hugs’* on every thread on C2. Literature has documented that feelings of loneliness and isolation are factors that could contribute to NSSI [52] and it is possible that the creation of a community spirit by enabling automatic comments could help alleviate some of these negative feelings. In addition to this, the domain *Sarcasm and Self-Deprecation* occurred 59 times on C2 and just 8 times on C1. Sarcasm has been previously described as a form of humour where the speaker attempts to send across a meaning that is opposite to the literal statement being uttered [53]. On the other hand, self-deprecation is a style of jocularity that is self-defeating and serves to bolster interpersonal relationships at the cost of self [54]. Indeed prior literature found that contextual humour that offers a relatability with self-defeating memes allowed people to laugh at their challenges while enabling them to build a connection with others dealing with similar situations [55]. This is relevant due to the fact that prior literature on an online game community found the use of humour to act as a type of community building “cushion glue” used to connect and solidify the boundaries and identity of the online community [56]. On the other hand, in line with the above-reported hypothesis, “narrative reinforcement” could be more likely in this group by leading to the normalization and justification of self-harm due to the sharing of similar stories and life experiences [24]. In addition to this, research has indicated that self-defeating humour is associated with worse mental health conditions, and is linked to rumination, brooding and common suicidal ideation risk factors [54]. Therefore, it is possible that the sharing of narratives centred around venting, trauma, and irony on C2 could in turn reinforce self-harm among the readers of this community. Moreover, the irony and attention seeking that was prevalent in the units categorized as *Sarcasm and Self-Deprecation* seemed to normalize and romanticize the act of self-harm, which prior literature has indicated could further promote self-harm behaviour [57].

Existing literature supports most of the domains that the coders categorized. For instance, in line with our domain *Addiction and Relapse Cycle*, there is growing evidence across research that NSSI has the potential to be considered as an addictive behaviour [58]. Authors from another study [22] found that people would often enquire whether their self-harm was valid, in line with the domain categorized as *Self-Worth and Validation through Self-Harm*. One study found that a portion of posts on message boards about self-harm centred around the theme Hiding/Shame [59] and another study with people who self-harm found mixed emotions with regard to scars and their changing physical presence [60]; not surprisingly, these aspects were found in our study within the domain categorized as *Concealing and Managing Scars*.

While the differences across the two communities, C1 and C2, may seem to be macroscopic and largely apparent, a deeper investigation points towards subtle differences at a more granular level that are essential to explore further. For instance, the themes that C2 spoke about tended to be more negatively loaded and conveyed more distress when compared to C1. Hence, it was expected that C2 would be marked with a more negative tone due to the leniency in group rules and the lack of moderation by administrators. Indeed, prior work has shown that moderation practices actively shape and enable what forms of expression are permitted in online health communities, often compelling members to conform while constraining alternative voices [61]. However, in contrast to expectations, during the sentiment analysis, it was found that the threads in C1 were marked with a more negative tone (significantly larger negative sentiment value) compared to C2.

It is helpful to interpret this unexpected finding within the broader communicative environments that shape interaction in C1 and C2. Each community sustains its own rhythms, norms of participation, and expectations regarding how distress is shared and responded to. Factors, such as moderation practices, the visibility and size of the group, platform affordances that shape how members in either group disclose personal experience, and historically accumulated habits of communication on both groups, could be utilized to interpret this result. The following section elaborates on how these different factors might have contributed to the higher level of negative sentiment found in C1.

First, in C1, stricter moderation and clearer posting rules may have encouraged users to make more direct, clinically oriented posts where one’s distress can be explicitly articulated, while in C2, apparent leniency in moderation could have led to a diverse set of expressive forms. This too is evident from the fact that the domain *Medical Care and Harm Management* was classified nearly 80 times for posts on C1 and only 22 times for C2, and it is likely that these texts contained more explicit distress-related information. Second, it is speculated that the text on the memes present on C2 contained most of the seemingly negative sentiment, which the sentiment analysis did not have access to, as it was embedded in the image. Indeed, in a paper by Dynel [62] on an analysis of COVID-19 memes, the authors stress the need for including multimodal voices to analyse discourse that merges both verbal and visual components. Hence, it is possible that our findings carried a multimodal blind spot.

Third, differences in community positioning could have also led to this finding. C1 is the larger and more publicly visible community, whereas C2 positions itself as a more insider, subcultural community. Therefore, it is likely that people in acute distress who are seeking immediate advice or help might choose to seek this primarily on C1. Instead, members on C2 might frequent this group more regularly, and hence, more acute expressions of distress may be rare.

Beyond moderation and structural differences, broader socio-cultural interpretations are also fitting. For instance, Ren and Ross [63] speak about the differences between sharing and self-disclosure within digital communities. They illustrate that while both involve the revelation of personal information, emotions, and experiences, sharing is inherently “audience-centric” and is governed by “platformed sociality”, whereas self-disclosure is inherently self-oriented, prioritizing the expression of the individual. The authors also draw on Gibson’s theory of affordances [64], suggesting that the features of a platform are not neutral but instead act as active gatekeepers on the nature and type of discourse that it contains. Hence, it could be said that the affordances that C2 provides to its members primarily foster a more performative sharing with reduced manifestations of one’s emotions, whereas in C1, members prefer to, and can engage in self-disclosure, thus leading to a higher overall negative sentiment.

This observation is strengthened by Bhatia’s work describing how communities on digital platforms represent a shared digital habitus of emotions, interests, and values [65]. Indeed, Ross and Bhatia [66] highlight how online communities act as digital repositories of previous interactions, posts, comments, and videos, thus reinforcing this digital habitus over time. Moreover, it is within such environments that users seek to present themselves as authentic members of the unique community for sociability and recognition. The negative sentiment observed in C1 may therefore be attributed to the perceived norms of authenticity embedded within this digital habitus. For instance, authors [67] have used the idea of pluralistic ignorance in the context of alcohol use on a university campus to indicate how incorrect misperceptions of a community norm could eventually influence individual behaviour. In the context of these NSSI e-Communities, literature has indicated that the most central appeal of these groups is the ability to maintain open, affect-laden, and honest conversations [29,68]. In this regard, a new member on C1 might align their tone to match this perceived community norm and hence utilize a more negative and self-disclosing style in order to affirm group belonging and signal authenticity.

Finally, from a clinical perspective and in line with the above, lower negative sentiment in C2 might not signal less distress, but instead the humour and irony may try to mask or even romanticize self-harm. Conversely, greater negative sentiment in C1 might not signal worse functioning, but rather a greater openness to signal distress and seek support [24,42].

In this respect, prior studies in other domains have demonstrated that social media can exist as a post for harm reduction and recovery [69], adding to this, the fact that a recent meta-analysis demonstrated how specialized treatments for self-harm are not more effective than treatment as usual [70], points toward the massive potential in leveraging social media to improve self-harm treatments. For instance, an aspect of the “person-centred framework” [71] stresses the importance of reflecting on the person’s precise language used by a person who self-harms in their interactions. The exploration of slang usage in our study attempts to provide a foundation for exactly that. Moreover, the creation of interventions that are more targeted, tailored, and able to account for these nuances that we learn from social media could improve the efficacy of treatments. Knowledge of these communities and self-harm group-based language could be valuable to clinicians and therapists. They could use the information to build quicker rapport and alliance. Moreover, it could be possible that knowledge of these intricacies could lead to quicker and more thorough disclosure from the patient. Thus, allowing clinicians to utilize a person-centred approach holistically. Furthermore, interventions for self-harm could explore the creation of a safe virtual space that contains the benefits of these e-Communities while minimizing its adverse aspects.

### 4.1. Methodological reflections

Beyond the findings discussed above, it is useful to reflect on the methodological choices made in this study and their implications. For instance, our unique slang-driven sampling strategy allowed us to capture conversations and experiences embedded in community-specific language practices. While this strategy provided us access to conversations that might have otherwise remained invisible to other researchers unfamiliar with insider terminology, it naturally excluded posts without slang, which may have sidelined certain other community-based interactions. Further, while coder immersion and the inductive content analysis were essential for identifying domains and sub-domains not anticipated a priori, and although strong inter-rater agreement added rigor to the process, this strategy also highlights the role of researcher subjectivity and the need for replication to improve the reliability of findings. Moreover, our choice of sentiment analysis illustrates both the potential and challenges of applying computational linguistic tools to such a study. While the NLTK package provides a structured approach to quantify emotional tone, existing lexicons are not optimized to account for slang or context-specific language (e.g., “yeet” or “styro”), making it likely that such terms are unrecognized by standard sentiment dictionaries.

Taken together, while the unique context in which our study was conducted required us to employ uncommon methodological approaches, our work also illustrates both the value and challenges of integrating these methods while studying sensitive online discourse.

### 4.2. Limitations

This study is not without its limitations, and building on these could be an avenue for researchers to explore in the future. Firstly, due to the unique data collection method (online, pseudonymous, and based on data scraping), demographic information about participants (e.g., age, gender, cultural background) could not be evaluated, which constrains the generalizability of our findings to broader populations. Secondly, the identity that users portray on the internet may not always reflect their offline realities, introducing the possibility that some analysed experiences may not fully align with lived contexts. Thirdly, assessing suicidal intent solely through text-based descriptions is inherently challenging; some posts may have described self-harm behaviour with underlying suicidal intent and therefore may not be strictly categorised as NSSI. Fourth, images and memes’ content were present only in one of the communities (C2). While we provide a descriptive coding of these materials in the Supplementary Material, systematic comparisons with C1 were not possible, and our sentiment analysis was limited to text-based descriptions. Finally, this study was not preregistered. While preregistration can be a useful tool, the exploratory nature of this work prioritized flexibility to follow emergent patterns.

Together, these limitations underscore the need for future research to adopt mixed-methods (e.g., including interviews, ethnographic approaches, etc), while also integrating multimodal analyses of texts and visuals. Visual content such as memes, screenshots or self-harm-related imagery could play a central role in shaping tone, humour and affective meaning in many online communities.

In this regard, future studies could employ ethically compliant approaches to analysing visual content, for instance, through the analysis of de-identified images or memes using the grammar of visual design [72] approach to examine how visual composition, salience, and framing convey affect and meaning. Researchers may also extract written text embedded within memes using optical character recognition techniques, allowing sentiment analysis of textual layers that are not otherwise captured. In addition, image style features such as colour palette, contrast, and visual themes could be annotated by independent coders. Finally, researchers could also employ multimodal deep-learning architectures [73] that combine textual and visual features to detect sentiment and humour.

Integrating such approaches could allow future work to capture forms of meaning-making that remain invisible within text-only analyses, reduce the multimodal blind spots inherent in traditional sentiment tools, and help balance both exploratory and confirmatory designs aims by providing a more holistic understanding of communication practices within NSSI e-Communities.

### 4.3. Conclusion

This study provides an in-depth insight into the language used in NSSI e-Communities and highlights intricate differences across different sites, which are linked to different expressive needs of their members. Owing to the growing popularity of bidirectional media and the growing significance of social media in people’s lives, this study is significant as it studies a new trend in the domain of NSSI. Thus, adding to a growing corpus that seeks to understand the nuanced existence of self-harm communities on social media. Moreover, a strong case is made for a deeper exploration into the functions that slang words serve for people in NSSI e-Communities, such as secrecy due to the fear of being discovered and building a sense of community through bonding over shared word meanings. It is apparent that these groups serve as a space for relatability and bonding over shared problems; however, future research and interventions must determine adaptive and healthy methods to sustain the spirit of belonging, while also encouraging behaviours like self-compassion and empathy. Finally, research could employ and integrate in-depth approaches, such as interviews with community moderators, virtual ethnography, community-engaged data collection, and longitudinal observations to deepen understanding of both the value these communities hold for people who self-injure and the complexity of their NSSI behaviour.

## Supporting information

S1 File Supplementary Images and Description of Memes. (DOCX)
