# Supplementary material for: Inside the world of non-suicidal self-injury e-communities: Language, identity and need for belonging
Source: PLoS One. 2025 Dec 31;20(12):e0339975. doi: 10.1371/journal.pone.0339975 (PMC12755822; doi:10.1371/journal.pone.0339975)
Supplement: S1 File — (DOCX) [file pone.0339975.s001.docx]

**S1 Figure**

**
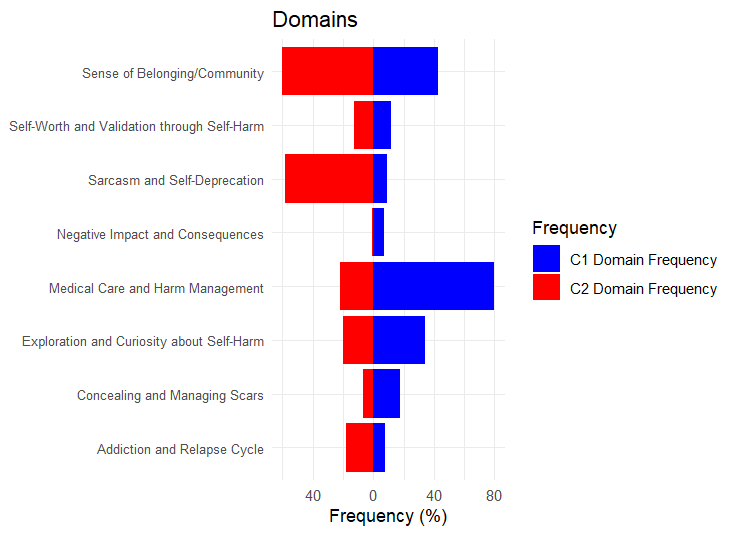
**

**S2 Figure**

**
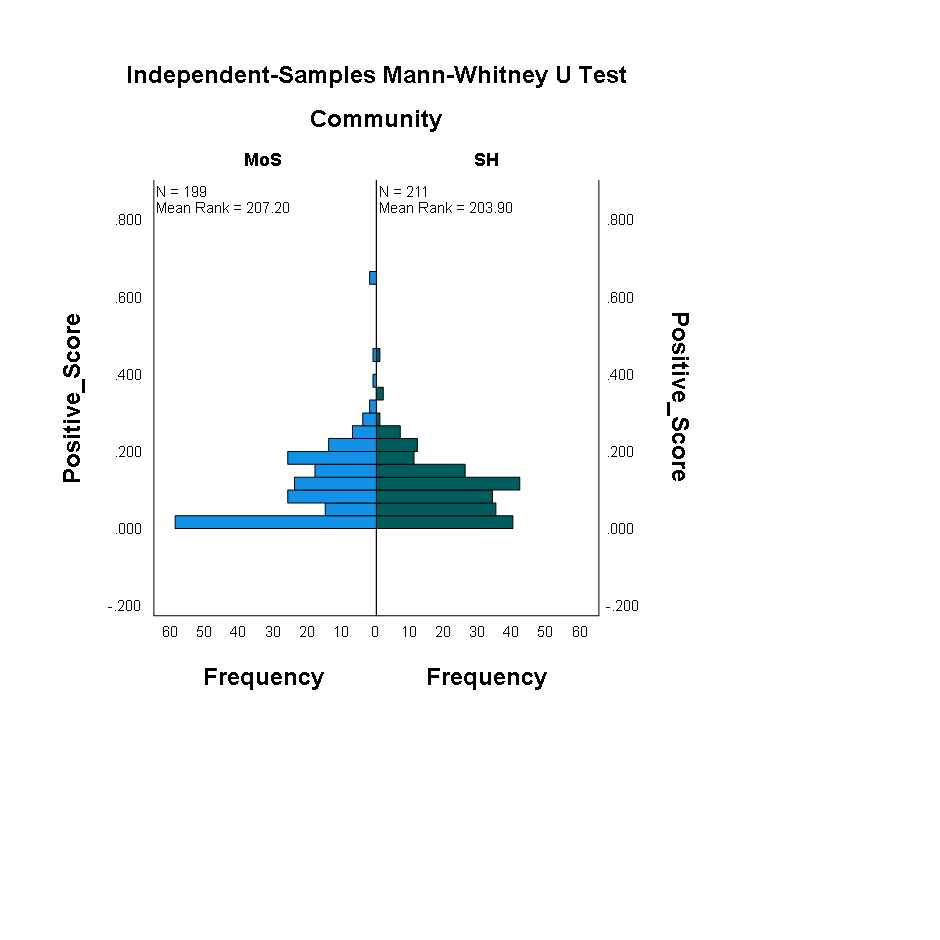
**

**S3 Figure**

**
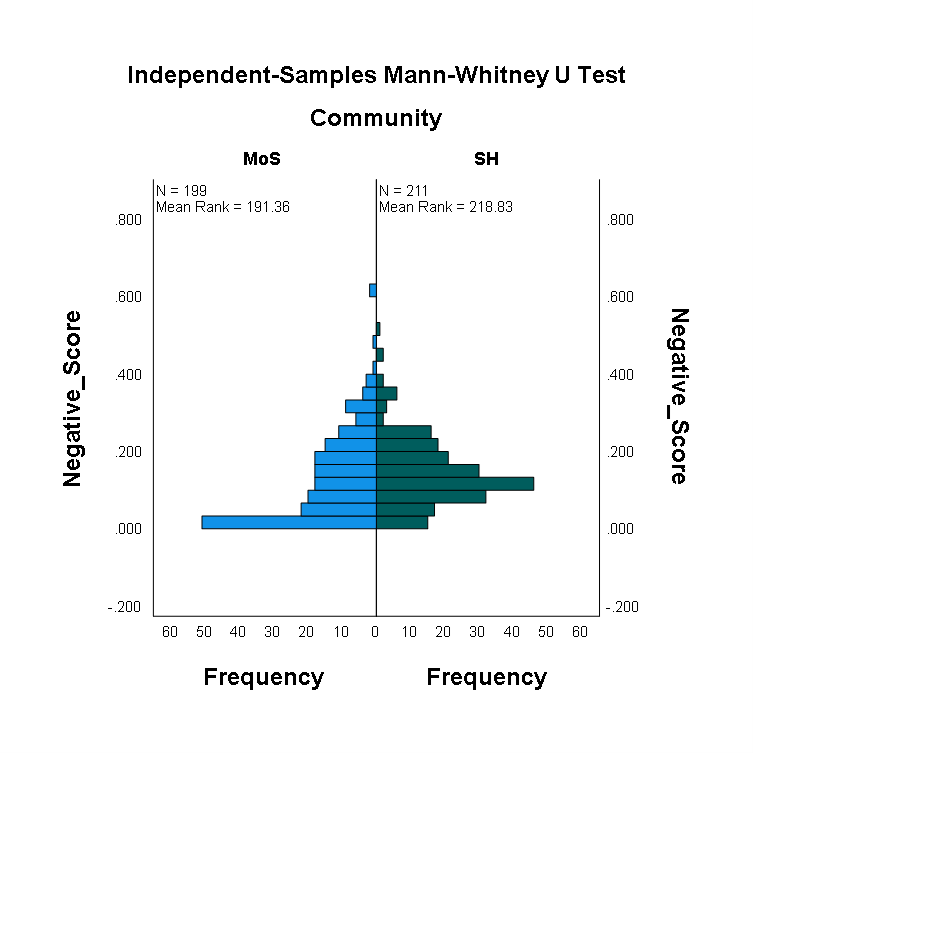
**

**S4 Figure**

**
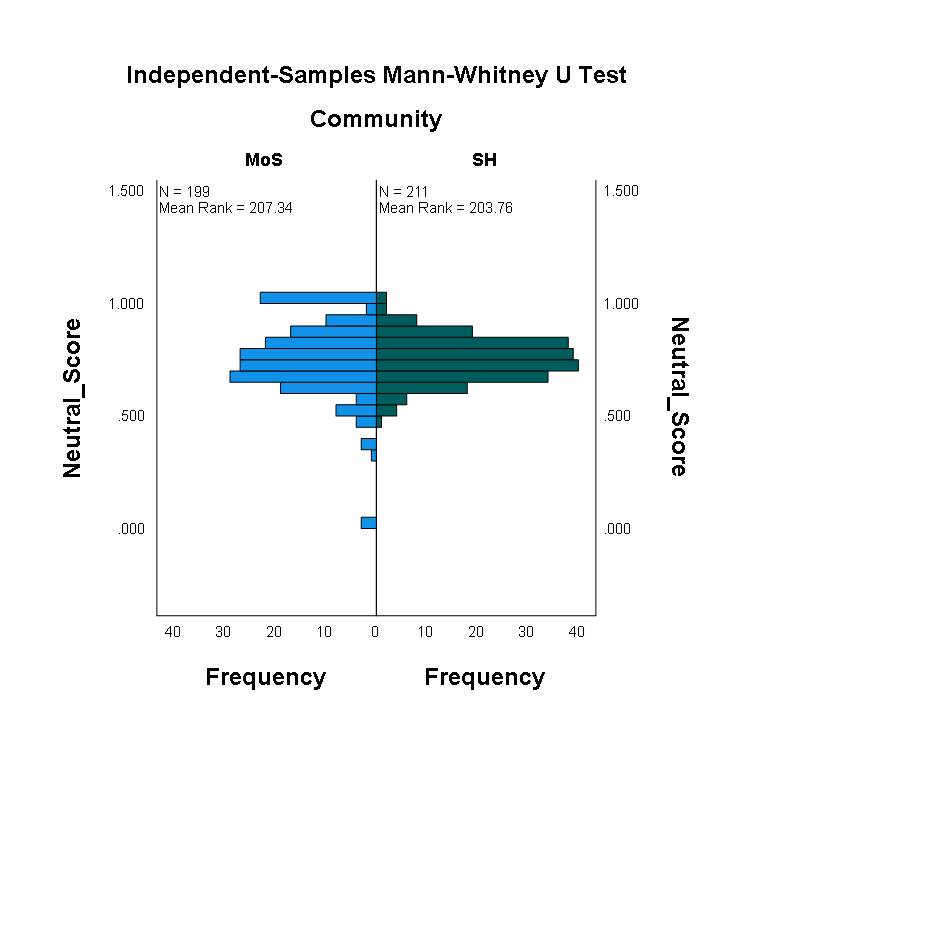
**

**S5 Figure**

**Supplementary Analysis: Meme and Image Content in C2**

Visual content was uniquely present in C2, where 113 image units (97 memes, 16 other images) were identified. Because C1 contained no images, no cross-community comparisons were possible. We therefore report a basic descriptive coding of image units on C2 down below. Of the 113 images, 97 were classified as memes and 16 were classified as images; 40 units contained direct or indirect references to self-harm, and only 6 units contained trigger warnings for references to self-harm.

All the 16 units that were classified as images were categorized within the domain *Symbols representing Hope,* as they all represented things that brought positivity, hope, and implicitly signalled mental health recovery. On the other hand, all other units that were classified as memes had varying emotion types that were predominantly negative: anger, loneliness, fear, frustration, confusion, and feelings of overwhelm. Moreover, these memes were also classified for the predominant humour type. This was considered necessary as it was evident that the humour type was used as a vehicle to deliver the underlying emotion felt. Humour types included sarcasm, irony, tongue-in-cheek humour, self-deprecation, puns, absurdity, and exaggeration.

Domains: Overall, four major domains were identified:

- *Representation of Self:* Memes that diminish, mock, or ridicule the self, often with a self-deprecatory humour
- *Self-Harm Tendencies:* Memes that are direct references to self-harm behaviours and experiences.
- *Representation of Addiction:* Memes framing self-harm as an addictive process, including tolerance, urges, and relapse.
- *Symbols representing Hope:* Images depicting positivity, resilience, or recovery.

Domains and sub-domains frequency are reported in Table S1.

**Table S1**

| **Domains (N)** | **Subdomains (N)** |
| --- | --- |
| Representation of Self (51) | Minimising One’s Sense of Self (17) |
|  | Self-deprecatory (13) |
|  | Mocking one’s actions (12) |
|  | Poor mental health (9) |
| Self-Harm Tendencies (30) | Exaggerating one’s self-harm behaviour (11) |
|  | Feelings after self-harm (9) |
|  | Everyday situations (6) |
|  | Explicit Self-Harm references (4) |
| Representation of Addiction (16) | Severity of Addiction (7) |
|  | Overwhelming urges to self-harm (4) |
|  | Abstaining from self-harm by doing other harmful activities (3) |
|  | Addiction in everyday contexts (2) |
| Symbols representing Hope (16) | Comfort through nature and animals (7) |
|  | Creative Projects (6) |
|  | Childhood toys as symbols of safety (3) |
